# Noninvasive Imaging of Ras Activity by Monomolecular Biosensor Based on Split-Luciferase Complementary Assay

**DOI:** 10.1038/s41598-017-08358-3

**Published:** 2017-08-30

**Authors:** Liang Chen, Wei Bing Leng, De Zhi Li, Hong Wei Xia, Min Ren, Qiu Lin Tang, Qi Yong Gong, Fa Bao Gao, Feng Bi

**Affiliations:** 1 Laboratory of Molecular Targeted Therapy in Oncology, State Key Laboratory of Biotherapy, Sichuan University, Chengdu, Sichuan China; 2 Department of Medical Oncology, West China Hospital, Sichuan University, Chengdu, Sichuan China; 3 Department of Radiology, West China Hospital, Sichuan University, Chengdu, Sichuan China

## Abstract

Deregulated activity of Ras GTPases has been observed in many types of human cancers, and contributes to the diverse aspects of carcinogenesis. Although the significance in tumorigenesis has been widely accepted and many therapeutic drugs are under development, little attention has been dedicated to the development of sensors for the Ras activity *in vivo*. Therefore, based on the split *firefly* luciferase complementation strategy, we developed a monomolecular bioluminescent biosensor to image endogenous Ras activity in living subject. In this biosensor, two inactive luciferase fragments are sandwiched by Raf-1, whose conformation changes upon GTP-Ras binding. Thus, the Ras activity can be surrogated by the intensity of the complementary luciferase. The bioluminescence analyses demonstrated that this novel biosensor behaved the robust and sensitive reporting efficiency in response to the dynamical changes of Ras activity, both in living colorectal cancer cells and *in vivo*. Compared to the traditional method, such as the pull-down assay, the bioluminescent sensor is simply, noninvasive, faster and more sensitive for the analysis of the endogenous Ras activity. This innovative work opens up the way for monitoring the preclinical curative effect and high-throughput screening of therapeutic drugs targeting Ras pathways.

## Introduction

The Ras proteins function as nucleotide-dependent switches and control many signaling pathways that are key regulators of cellular proliferation, differentiation, survival, and tumor malignant transformation. Deregulation of Ras pathways has been observed in a variety of human tumors and is essential for the genesis and maintenance of the tumoral phenotype. Targeting Ras signaling pathways might inhibit tumor growth, survival and spread. Several of therapeutic agents are showing promise in the clinic and many more are under development due to new approaches of drug discovery and new insights into Ras function^1, 2^. Molecular imaging has emerged as a sensitive technology to advance our understanding of disease mechanisms at the molecular level and accelerate drug discovery and development. The objective of our work is to develop a sensor for imaging Ras signaling activity, in order to facilitate the identification of therapeutic compounds and noninvasively evaluate their efficacies in preclinical models.

In recent years, research efforts have been dedicated to the visualization of Ras activity in living cells using Fluorescence resonance energy transfer (FRET) analysis, revealing the spatiotemporal dynamic regulation of Ras activity^3–8^. Furthermore, under the two-photon fluorescence lifetime imaging microscopy, FRET-Ras sensors (FRas2 sensors) were sensitive to Ras activation in cortical neurons^7^. However, the weaknesses of FRET assay, such as auto-fluorescence, low sensitivity, and challenges for stable expression, raise doubts about its suitability for *in vivo* application and the therapeutic efficacy analysis^9^. As an alternative, luciferase fragment complementation assay (LFCA) is another approach to image molecular signaling events in living animals, which can circumvent cell and tissue auto-luminescence and improve the signal to noise ratio. The basis of this bioluminescent assay is that the luciferase enzyme can be split into two fragments without enzymatic activity, N-terminal and C-terminal (NLuc and CLuc). When the NLuc and CLuc fragments are brought into close proximity by the protein-protein interaction or the molecular conformation change, they are able to reconstruct luciferase enzyme activity. Using this strategy, we have successfully developed a series of novel bioluminescent biosensors for imaging the activities of Rho GTPase and Src Tyrosine kinase in live subjects^10, 11^. In this study, we introduce another novel monomolecular bioluminescent biosensor (Raf-Fluc) to image endogenous Ras activity in living subjects. Ras proteins function as intracellular molecular switches, cycling between the GDP-bond (inactive) and GTP-bond state (active). Although the significance in tumorigenesis has been widely accepted, little attention has been dedicated to the development of sensors for the Ras activity, especially *in vivo*. Raf-1 protein is a specific direct effector of Ras proteins and connects upstream tyrosine kinases and downstream serine/threonine kinases. Previous biochemical analyses have identified that Raf-1 adopted two conformations: open active and closed inactive^12, 13^. We presumed that LFCA strategy could be applied to monitor the conformational changes of Raf-1, which act as the surrogate for the Ras activity.

## Results

### Design of the genetically encoded monomolecular Ras sensor based on spilt luciferase complementary assay

**Figure 1:**
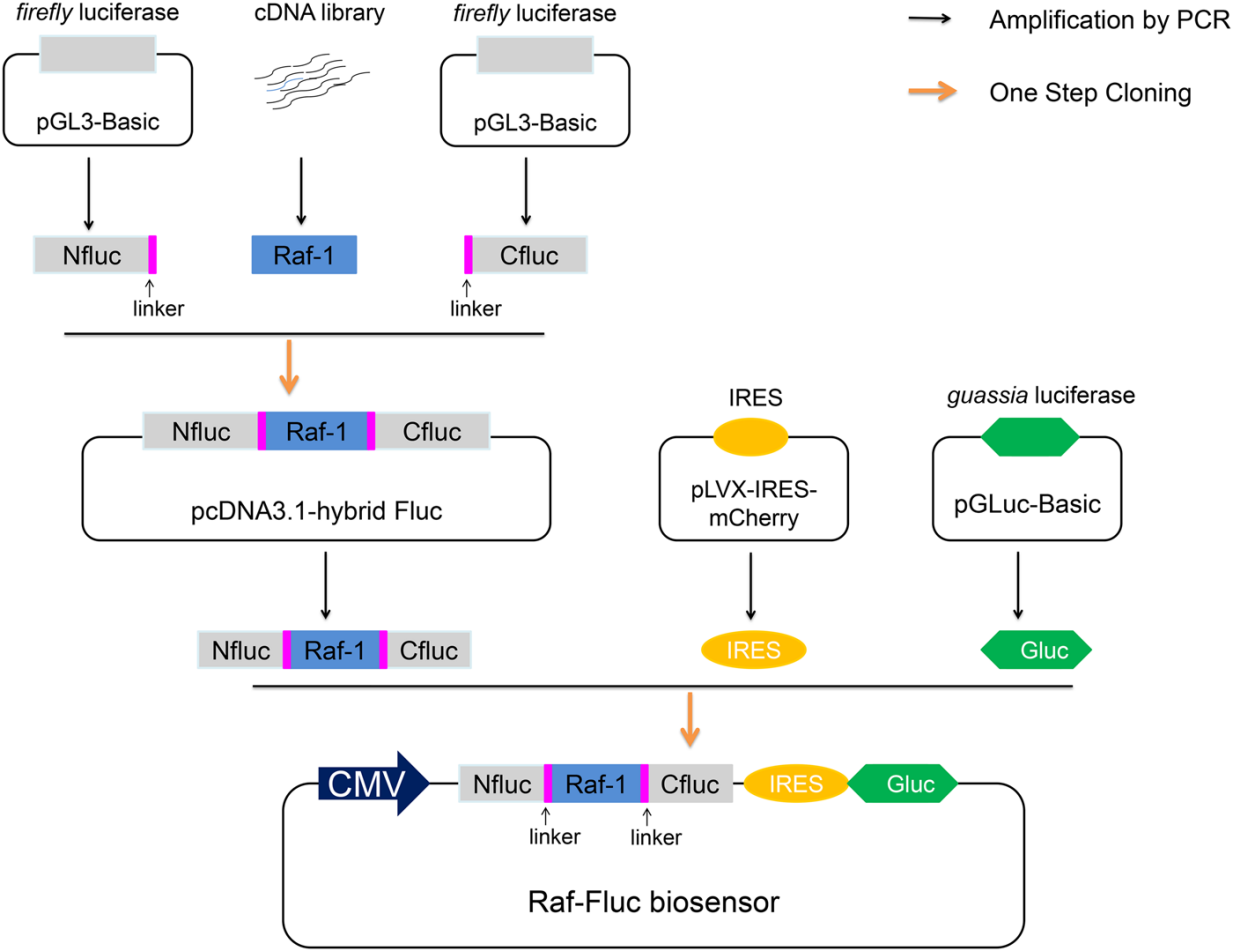
The construction and structure of the Raf-Fluc. In the flow chart, the fragments were amplified by PCR from the indicated plasmids or the cDNA library of K562 cell line, and seamlessly constructed into pcDNA3.1(+) using ClonExpress^TM^ MultiS One Step Cloning kit. The NLuc and CLuc fragments of split *firefly* luciferase were respectively fused to the N- and C-terminals of Raf-1, through short flexible linkers (G_2_S)_4_ or (G_4_S)_2_. The hybrid *firefly* luciferase (hybrid Fluc) and *gaussia* luciferase (Gluc) were coexpressed via the IRES under the control of the CMV promoter.

**Figure 2:**
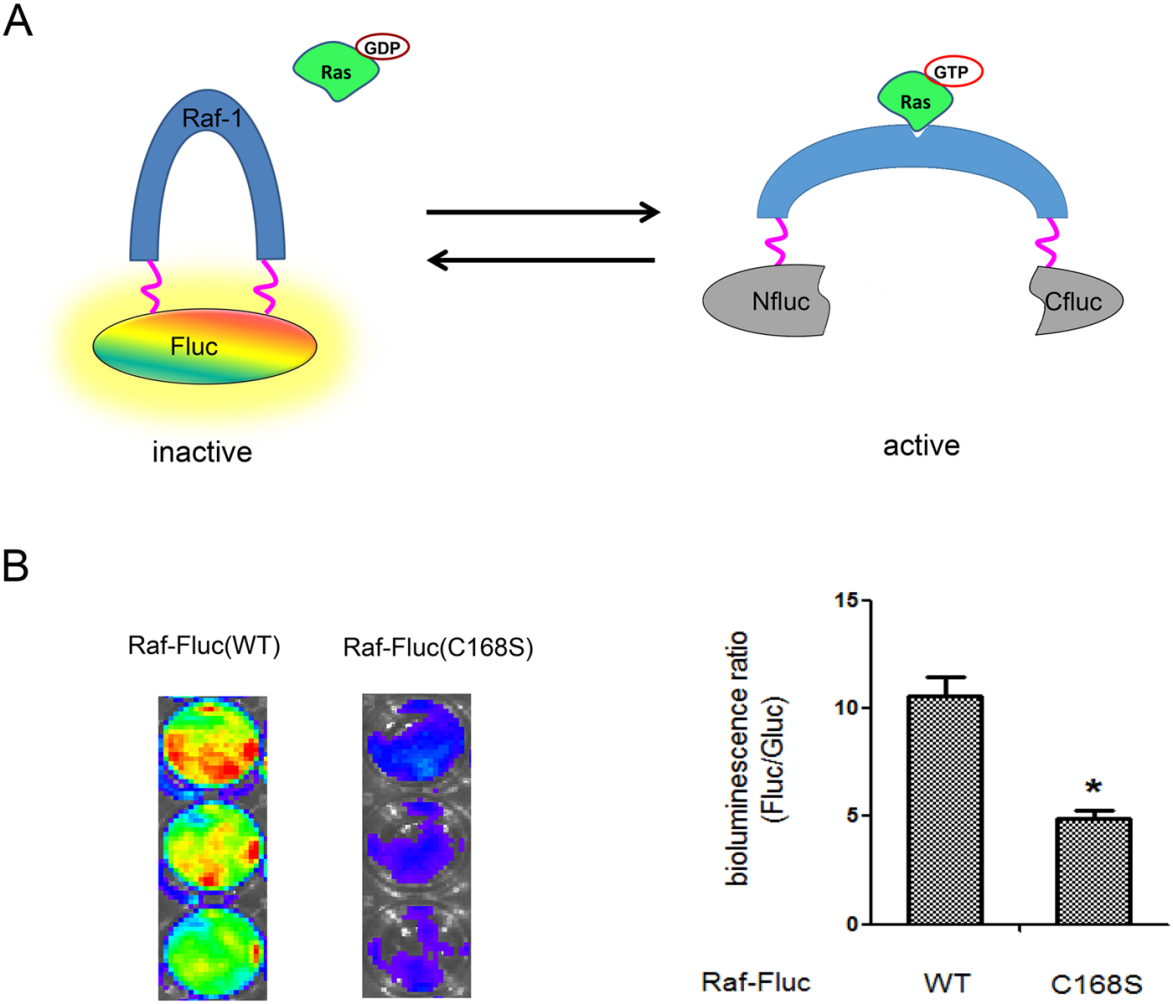
Schematic strategy of imaging Ras activity via detection of Fluc complementary assay. (**A**) The schematic diagram of the Raf-Fluc. In the GDP-bound state (inactive), Raf-1 is present as an inactive closed conformation, the fragments of *firefly* luciferase are brought into closer proximity and restore luciferase activity. Upon Ras switching from GDP-bound (inactive) to GTP-bound (active) form, the GTP-Ras binds to the Raf in the sensor and induces conformational changes, sterically preventing the reconstitution of the functional luciferase. (**B**) Comparison of the complementary bioluminescence intensity between the wild-type and negative mutant control in SW1116 cells. Data are expressed as the mean ± S.D. of triplicate determinations. Three additional experiments gave similar results. Asterisk (*) indicates significant difference from the wild-type sensor by test (*p* ≤ 0.05).

This novel bioluminescent Ras sensor takes advantage of the fact that the GTP-Ras (active) can induce Raf-1 to transit from a closed to an open conformation. In this strategy, the *firefly* luciferase was rationally dissected into two non-functional fragments (Nluc 1-416aa and Cluc 398-550aa)^14^, and a hybrid luciferase (hybrid-Fluc), in which Nluc and Cluc fragments were respectively fused to the N- and C-terminals of Raf-1, was generated. The short flexible linkers (G_2_S)_4_ or (G_4_S)_2_ were inserted at the junctions between Raf-1 and luciferase fragments to minimize the steric hindrance on luciferase complementation efficiency (Fig. 1). In the GDP-bound state (inactive), Raf-1 is present as an inactive closed conformation, the fragments of *firefly* luciferase are brought into closer proximity and restore luciferase activity. In cellular response to upstream tyrosine kinase stimuli such as growth factor, Ras proteins switch from GDP-bound (inactive) to GTP-bound (active) form. The GTP-Ras binds to the Raf in the sensor and induces conformational changes, which yields an opened structure, sterically preventing the reconstitution of the functional luciferase (Fig. 2A). Thus, this Ras biosensor is designed to increase bioluminescent activity following the inhibition of Ras activity, and more suitable for investigating the efficiency of Ras signaling targeted therapy.

Apart from the hybrid *firefly* luciferase, the bioluminescent Ras biosensor also constitutes some other essential elements: (a) the internal ribosome entry site (IRES), which is the sequence that can recruit ribosome and then initiate the translation of downstream gene sequence^15^. (b) *Gaussia* luciferase (Gluc), which is the smallest known coelenterazine-using luciferase (Fig. 1). By this way, the *gaussia* luciferase, as the internal control, was co-expressed with the hybrid *firefly* luciferase via the IRES, to balance the efficacy of transfection and expression of the biosensor among the different treatments. Thus, we can estimate and compare the activity of Ras by measure the normalized bioluminescence ratio of *firefly* luciferase and *gaussia* luciferase (Fluc/Gluc).

To corroborate the reporting abilities of luciferase in response to Raf-1 conformation changes in the sensor, the biosensor harbored Raf-1(C168S) mutation [Raf-Fluc(C168S)] was constructed as a negative control. This mutation disturbs the intra-molecular binding of CR1 to the CR3 kinase domain and keeps Raf-1 adopt an open conformation regardless of the Ras status^13^. As shown in Fig. 2B, we transfected either the Raf-Fluc(WT) or Raf-Fluc(C168S) into SW1116 cells, respectively. As expected, 24 h after transfection, the bioluminescence of Raf-Fluc(C168S) mutant was much lower when compared to the Raf-F1uc(WT). The results showed significant difference by test with SPSS 14.0 for Windows software (SPSS Inc) (p ≤ 0.05). These results confirm that the complementary bioluminescence intensity is dependent on the conformations of the Raf-1 in the sensor, thus implying the responsive ability of the Raf-Fluc to the Ras status.

### Dynamic imaging of the Raf-Fluc in response to Ras signaling in living cells

EGFR-Ras signaling pathway is one of the most recognizable Ras signaling, and many targeted therapy drugs have been applied in clinic, such as Gefitinib and Cetuximab. We next checked the performance of the Raf-Fluc in response to EGFR-Ras signaling in living cells by administering EGF or Gefitinib to activate or inhibit this pathway.

**Figure 3:**
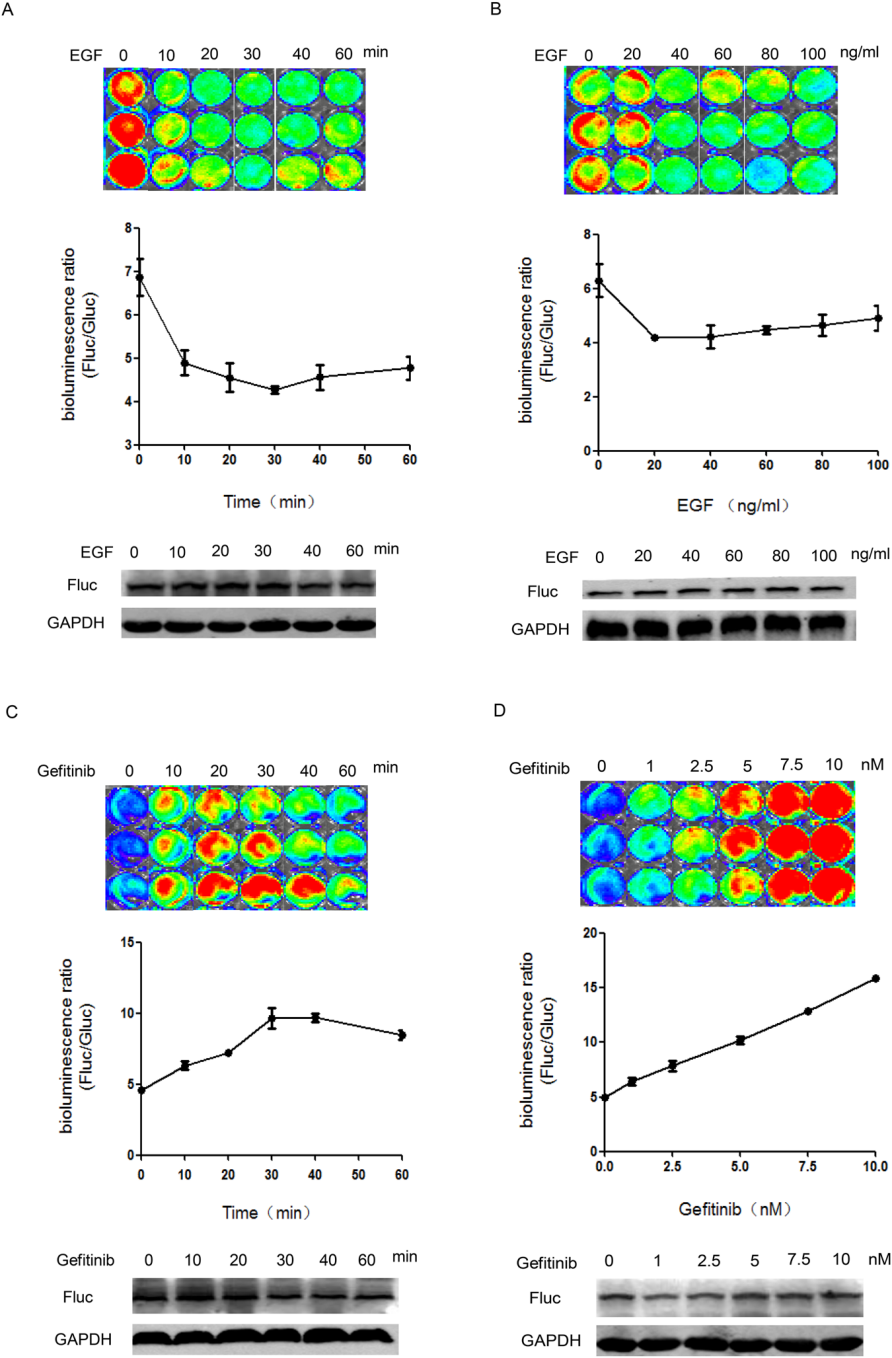
Dynamic imaging of the Raf-Fluc in response to Ras signaling in living cells. The normalized bioluminescence ratio was calculated by Fluc/Gluc. Values are mean ± S.D. of three separate experiments. Western blotting was performed under the corresponding conditions to confirm the specificity of the sensor. (**A**) EGF-induced, time-dependent analyses of the Raf-Fluc in living SW1116 cells. 40 ng/ml EGF was administered after starving overnight. (**B**) Dose-response curve of EGF-induced bioluminescence changes in SW1116 cells. After starving overnight, different concentrations of EGF were incubated for 30 min. (**C**) Gefitinib-induced, time-dependent analyses of the Raf-Fluc in living SW1116 cells. 5 nM Gefitinib was administered to inhibit Ras signaling pathway for different times. (**D**) Dose-response curve of Gefitinib-induced bioluminescence changes in SW1116 cells. 0–10 nM Gefitinib was administered to block Ras signaling for 30 min.

For the time-dependent assay of EGF stimulation, Raf-Fluc-SW1116 cells, which were transfected the Raf-Fluc, were serum-starved overnight and stimulated with 40 ng/ml of EGF for the indicated times. The results indicated that 40 ng/ml EGF induced a remarkable decrease of luciferase activity within 10 min (about 29%), and reached to its maximum response within 30 min (about 38%) (Fig. 3A). In contrast, Raf-Fluc(C168S)-SW1116 cells showed little changes in the bioluminescence activity (data not shown). For the dose-dependent assay, Raf-Fluc-SW1116 cells were serum-starved overnight and incubated with increasing concentrations of EGF for 30 min. The data showed that the luciferase activity of the Raf-Fluc reached the highest after 20–40 ng/ml EGF stimulating (Fig. 3B). The results demonstrate that the Raf-Fluc is sensitive to measure the activation of Ras activity induced by EGF.

On the other hand, Gefitinib, a small molecular inhibitor of EGFR, was administered to inhibit the activity of Ras signaling. In time course studies, Raf-Fluc-SW1116 cells were pretreated with 5 nM Gefitinib at various times. As indicated in Fig. 3C, 0.5 nM Gefitinib induced a remarkable luciferase activity increase in a time-dependent manner, and the activity of Ras proteins was mostly inhibited by Gefitinib at 30–40 min. In dose response studies, as shown in Fig. 3D, Raf-Fluc-SW1116 cells were treated with increasing concentrations of Gefitinib for 30 min. The luciferase activity appears to be increasingly higher as the concentration of Gefitinib. These results confirm that the Raf-Fluc is sensitive to monitor the inhibition of EGFR-Ras signaling induced by Gefitinib.

In order to further confirm that the bioluminescence changes were not due to the different expressions of *firefly* luciferase protein, we analyzed the expression levels of *firefly* luciferase using western blotting. The band densities of the western blot images were analyzed using analysis of variance (ANOVA) with SPSS 14.0 for Windows software (SPSS Inc). These results indicated that there was no obvious difference in the protein expression among different treatments (P ≤ 0.05). These results further confirm that the Raf-Fluc is specific for Ras signaling pathway and that the observed change of luciferase activity was induced by the Ras signaling dependent conformation changes.

### The Raf-Fluc was a sensitive and reversible modality to detect Ras signaling

**Figure 4:**
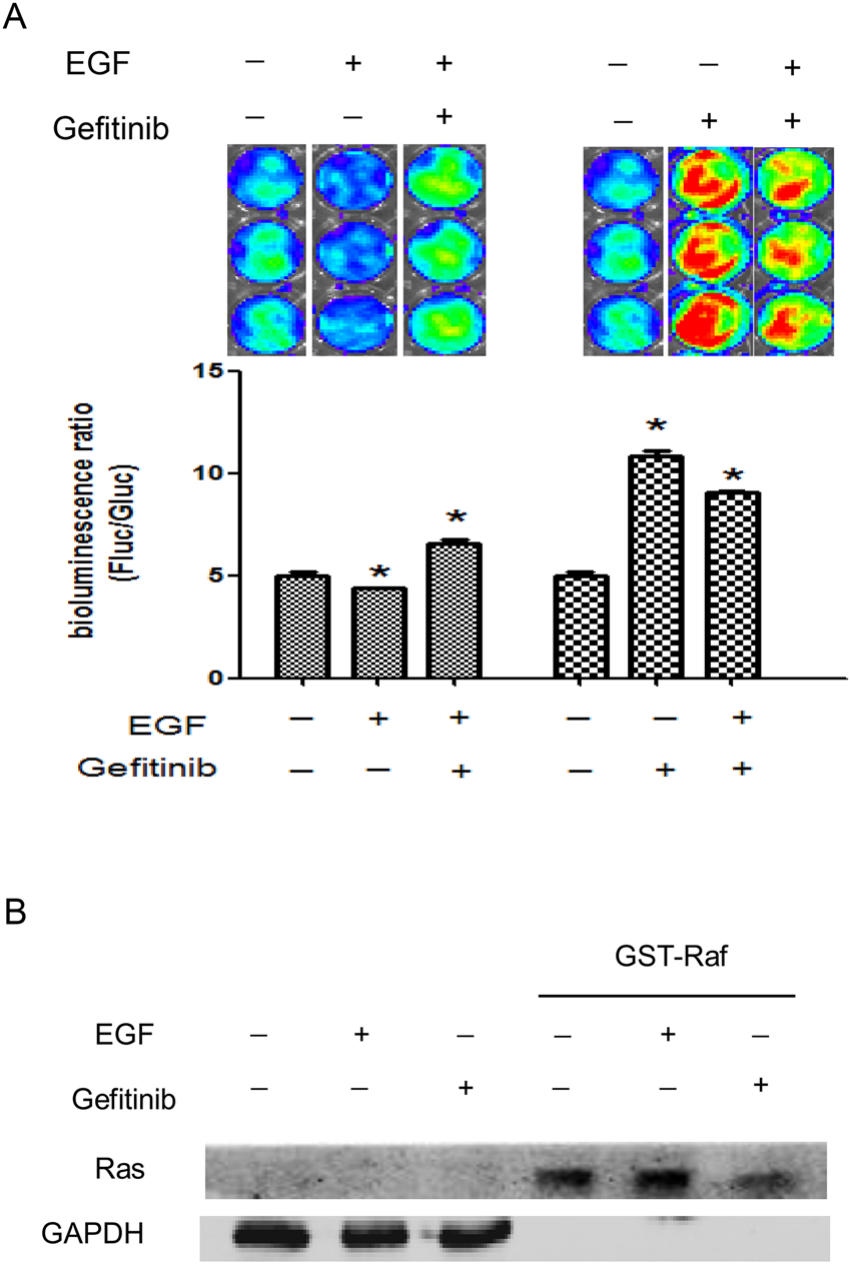
The Raf-Fluc was a sensitive and reversible modality to detect Ras signaling. (**A**) Bioluminescence analyses of the Raf-Fluc in response to the antagonism treatments. The Raf-Fluc-SW1116 cells were respectively performed pretreatment with 40 ng/ml EGF or 5 nM Gefitinib for 30 min, and then 5 nM Gefitinib or 40 ng/ml EGF were correspondingly administered for 30 min to reverse the pretreatment. And the treatment with vehicle served as the control. Values are mean ± S.D. of three separate experiments. Asterisks (*) denote samples that show a difference from the vehicle control with statistical significance by analysis of variance (ANOVA) (*P* ≤ 0.05). (**B**) pull-down assay was used to measure the activity of Ras proteins after EGF or Gefitinib administration. GAPDH was used as a loading control.

As we known, unlike the fluorescence complementation, luciferase complementation does not assemble irreversibly^11^. After confirming the effect of EGF and Gefitinib on the activity of Ras signaling, we further explored whether the changes of luciferase activity could be reverted by the antagonism treatment. To test the reversibility of the Raf-Fluc, the Raf-Fluc-SW1116 cells were respectively performed pretreatment with 40 ng/ml EGF or 5 nM Gefitinib for 30 min, and then 5 nM Gefitinib or 40 ng/ml EGF was correspondingly administered for 30 min to revert the pretreatment. And the treatment with vehicle served as the control. The results showed that differences from the vehicle control with statistical significance by analysis of variance (ANOVA) (P ≤ 0.05), indicating the Raf-Fluc could reveal the following reversion events of the activity of Ras signaling (Fig. 4A). In another words, the Raf-Fluc could monitor the Ras activity in real-time, thus allowing for the detection of kinetic of Ras signaling.

In order to further demonstrate that the bioluminescence reflected the real activity change of Ras signaling, we used traditional pull-down assay to measure the activity of Ras proteins after EGF or Gefitinib administration. The results indicated that the change of Ras activity was consistent with the results revealed by the bioluminescent analyses (Fig. 4B). Furthermore, the bioluminescence imaging is more sensitive and convenient compared with the pull-down assay.

### The Raf-Fluc was sensitive to distinguish different mutations of H-Ras

**Figure 5:**
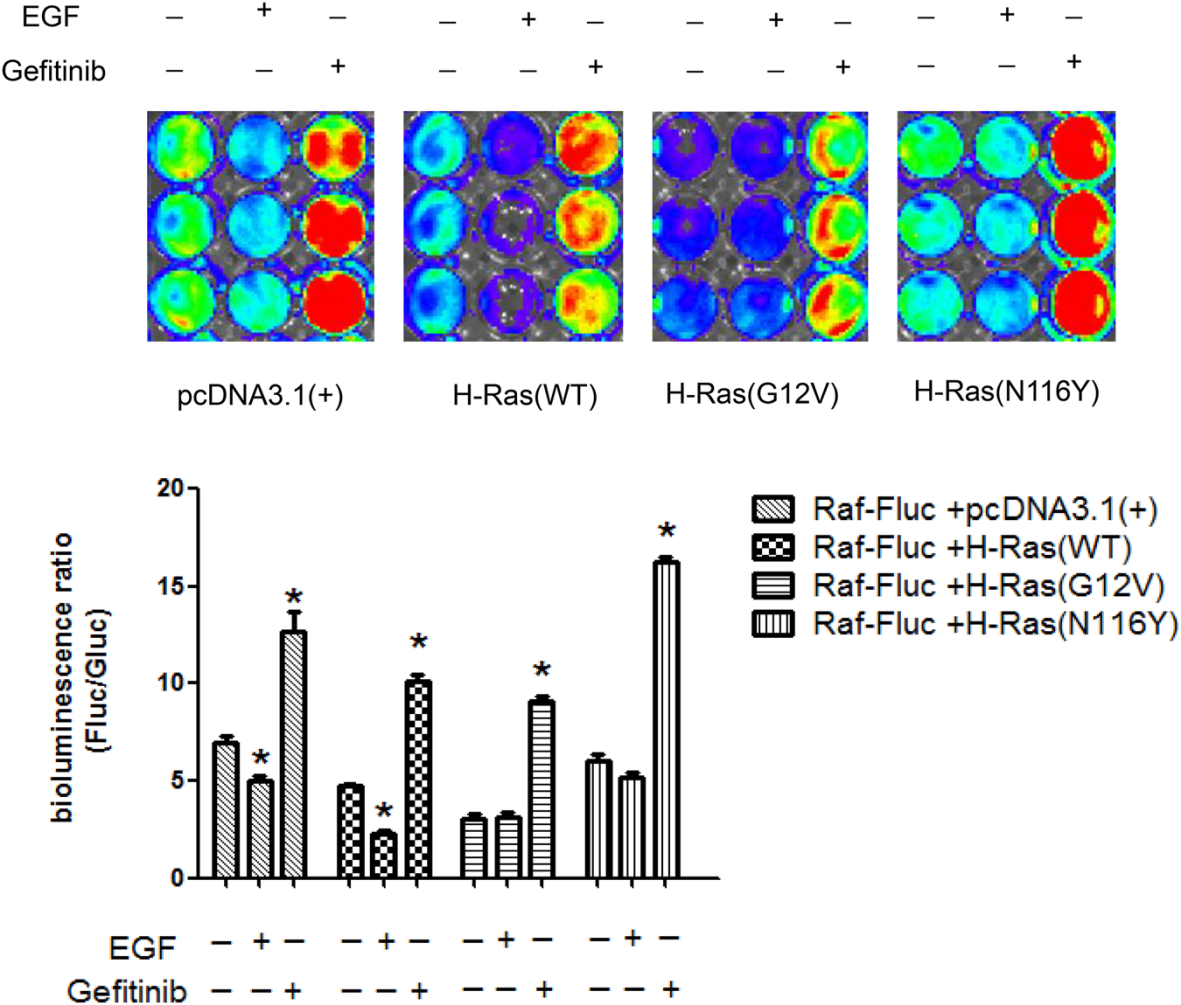
The Raf-Fluc was sensitive to distinguish different mutations of H-Ras. The cotransfected cells incubated with 40 ng/ml EGF or 5 nM Gefitinib for 30 min. The normalized bioluminescence ratio was calculated by Fluc/Gluc. Values are mean ± S.D. of three separate experiments. Asterisk indicates significant difference from the vehicle control.

Subsequently, we aimed to demonstrate that the Raf-Fluc could respond to and distinguish different mutations of H-Ras. For that purpose, the positive mutation H-Ras(G12V)^16^ and the negative mutation H-Ras(N116Y)^17^ were generated from pcDNA3.1-H-Ras(WT) plasmid by site directed mutagenesis. The cotransfected SW1116 cells [respectively with H-Ras(WT), H-Ras(G12V), H-Ras(N116Y) or pcDNA3.1(+) vector], were treated with 40 ng/ml EGF or 5 nM Gefitinib for 30 min. As indicated in Fig. 5, the cells transfected with H-Ras(G12V) appeared the lowest luciferase activity and were responsive to Gefitinib treatment, but not to EGF. On the contrary, cells transfected with H-Ras(N116Y) behaved the highest luciferase activity. And cells transfected with H-Ras(WT) showed more sensitive to EGF treatment compared to the pcDNA3.1 control. This phenomenon could be deciphered by that the exogenous H-Ras(WT) increased the ‘base level’ of Ras proteins. Altogether, these results demonstrate that the Raf-Fluc is sensitive to distinguish different mutations of H-Ras.

### The application of the Raf-Fluc to monitor Ras signaling *in vivo*

**Figure 6:**
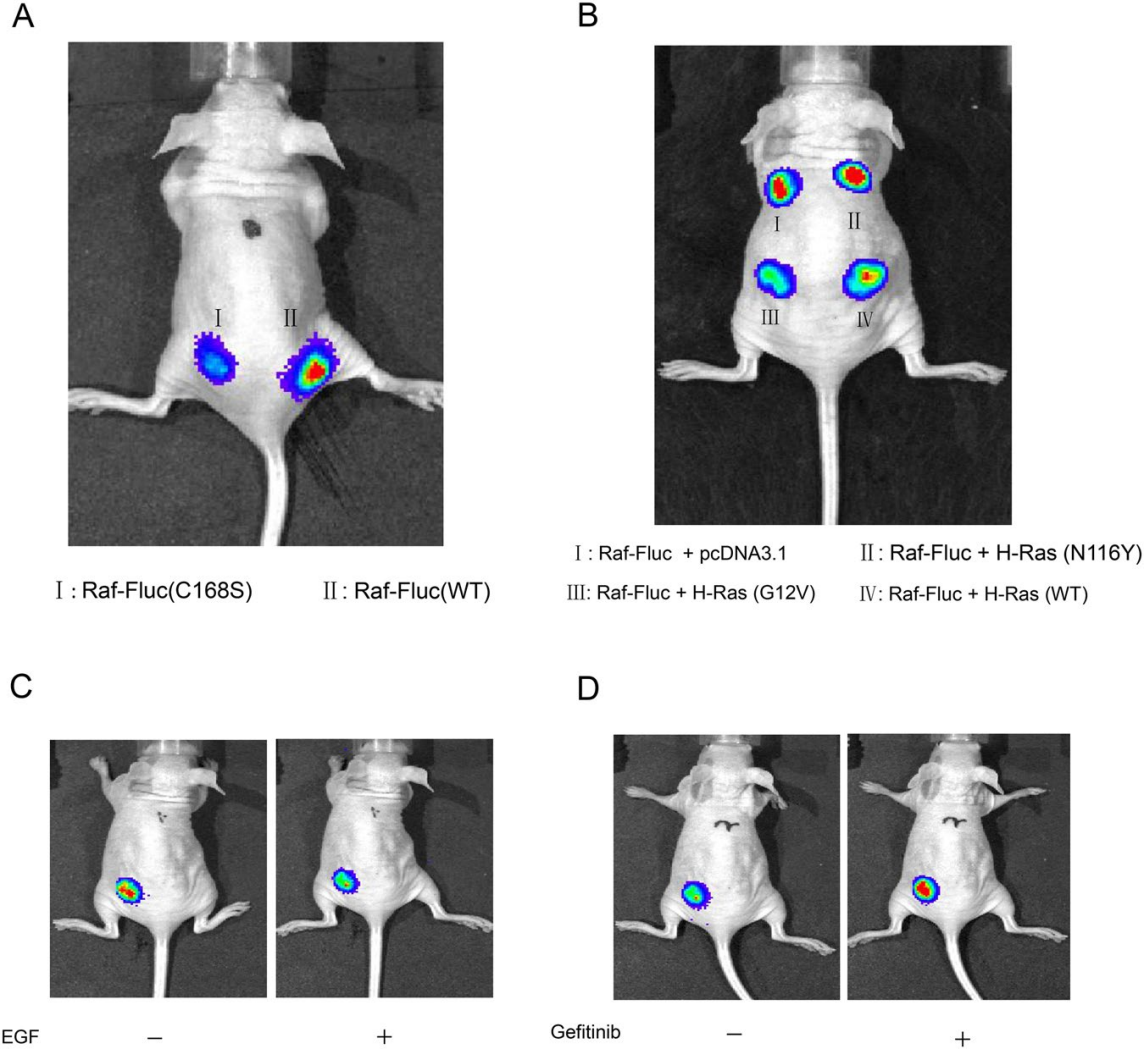
The application of the Raf-Fluc to monitor Ras signaling *in vivo*. (**A**) Comparison of the bioluminescence intensity between the Raf-Fluc(WT) and Raf-1-Fluc(C168S) *in vivo*. (**B**) The Raf-Fluc was sensitive to distinguish different mutations of H-Ras *in vivo*. The SW1116 cells were cotransfected with Raf-Fluc and H-Ras [H-Ras(WT), H-Ras(G12V), H-Ras(N116Y) or pcDNA3.1(+)], and implanted subcutaneously after 24 hours. (**C** and **D**) Bioluminescence analyses of the Raf-Fluc in response to the EGF or Gefitinib treatment *in vivo*. For the Raf-Fluc-SW1116 xenograft models, 10 ug/kg EGF or 50 mg/kg Gefitinib were injected intraperitoneally after a residence time of 24 hours. Bioluminescence was conducted by IVIS spectrum one hour later.

As is well-known, for the pharmacological drugs, the ability to modulate its target’s activity *in vivo* depends on multiple factors. However, up to now, it isn’t possible to measure the Ras activity *in vivo* due to the lake of appropriate technologies. We next investigated whether or not the *in vitro* performance could be replicated and measured in an *in vivo* environment. For that purpose, SW1116 cells harbored the sensor were subcutaneously injected into the flanks of nude mice to establish human tumor xenograft models. The *in vivo* bioluminescence imaging was conducted 24 h upon the injection. First, we compared the bioluminescence intensity between the wild-type [Raf-Fluc(WT)] and the mutant [Raf-1-Fluc(C168S)] *in vivo*. It is clear that the sensor remains her effectuality *in vivo* and the bioluminescence activity was significantly higher in the Raf-Fluc-SW1116 cells than the Raf-1-Fluc(C168S)-SW1116 cells (Fig. 6A). This indicates that the Raf-Fluc have great potential for *in vivo* application.

Then, we cotransfected the Raf-Fluc with exogenous H-Ras, as indicated in Fig. 6B, to further check whether the biosensor was sensitive to distinguish different mutations of H-Ras *in vivo*. The results showed that significant discrimination of luciferase activity was detected *in vivo* among different alleles of H-Ras as *in vitro* by analysis of variance (ANOVA) (*P* ≤ 0.05), which represent the different Ras activities. The xenograft with H-Ras(G12V) showed the lowest bioluminescence intensity, due to the more exogenous active H-Ras.

Finally, we further investigated whether the Raf-Fluc could be in response to the EGF or Gefitinib treatment *in vivo*. 10 ug/kg EGF or 50 mg/kg Gefitinib were injected intraperitoneally to xenografts with the Raf-Fluc after a residence time of 24 hours. Bioluminescence was conducted one hour later via a procedure as explained under experimental section. As Fig. 6C and D indicates, it is clear that the Raf-Fluc was significantly sensitive to the activity changes of Ras signaling induced by EGF or Gefitinib compared with the vehicle control. Taken together, these data demonstrated that the genetically encoded biosensor was able to measure the activity of Ras signaling *in vivo* and to investigate the efficacy of Ras signaling targeted therapy in preclinical cancer models.

## Discussion

The abnormal activity of Ras proteins is widely existed in human cancers, which is also associated with the treatment strategy, drug resistance and prognosis. The Ras signaling pathways have received considerable attention as a target for anticancer therapy due to its crucial role in carcinogenesis^18^. On the other hand, since the FRET based biosensor called ‘Ras and interacting protein chimeric unit’ (Raichu) was developed to image the spatiotemporal dynamics of Ras activity in single living cell^3^, the imaging of Ras protein is becoming a new research hotspot. However, little attention has been dedicated to the development of sensors for the Ras activity *in vivo*. And, it is difficult to employ these FRET based biosensors in living animals due to their high degree of auto-fluorescence and poor signal penetration depth through biologically heterogeneous tissues. LFCA is thought to have the most sensitive and highest dynamic range due to the enzymatic amplification of signals and the optimized biocompatible substrate (high cell permeability and high quantum yield) among all the protein-protein interaction (PPI) detection methods^19^. Several LFCA based reporters have been developed to visualize the activity of important molecules, such as Akt^20^, c-Myc^21^, Src^11^ and Rho GTPase^10^. But up to now, there is no bioluminescent biosensor for imaging the activity of Ras proteins, which functions as a crucial driver gene in many kinds of cancers. Here, we developed a novel monomolecular reporter for imaging the endogenous Ras proteins activity. Comparing with the bimolecular bioluminescent sensor reported by us to image Rho GTPase^10^, there was no need to introduce exogenous luciferase labeled Ras proteins, so this reporter system can reveal the intrinsic molecular events in living cells and *in vivo*. This work opens up new opportunities to investigate complicated biological processes of Ras signaling in living subjects, and have greater potency to accelerate drug discovery and development of Ras signaling inhibitors, and to evaluate drug efficacy in preclinical disease model.

It’s worth noting that the bioluminescent Ras reporter introduced in our work was constructed by sandwiching the full-length Raf-1 between the split luciferase fragments. The disadvantage is that its DNA sequences are too long to develop lentiviral vector. We initially considered to develop the sensor by flanking Nluc and Cluc fragment to the Ras-binding domain (RBD) of Raf-1, which have high affinity interaction with Ras. However, in our experience, this strategy showed little bioluminescence change upon Ras activation. This phenomenon could be deciphered by the low conformation change of RBD after interacting with GTP-Ras. Previous Raf-1 structure and function research have implied that 14-3-3 may suppress Raf-1 activity by binding to CR2 and CR3 of Raf-1 in a phosphorylation-dependent manner to maintain Raf in a closed conformation^22, 23^. The interaction of GTP-Ras with the RBD and CRD (cysteine-rich domain) of Raf alter the conserved 14-3-3-binding motif in CR2^24, 25^. This mechanism can explain why Raf-1 undergo conformation change upon Ras activation and binding. It has been reported that the R89L mutation within the RBD and the C168S substitution within the CRD completely eliminate Ras-dependent Raf-1 activation^26^. Interestingly, the R81L mutant and C168S mutant of Raf-Fluc biosensor had different bioluminescence in our experiment. The former had little change compared with the wild type. Whereas, the C168S mutant biosensor [Raf-Fluc(C168S)] displayed a significant decrease of bioluminescence, which was in agreement with a previous report stating that Cys168Ser mutation disrupts the intramolecular binding of CR1 to the CR3 kinase domain^13^. The conflicting and equivocal interpretations reconfirm the multiplicity of interaction of Raf-1 with Ras and the complexity of Raf-1 regulation.

Ras proteins function as important nucleotide-dependent switches by cycling between a GDP-bond state (inactive) and a GTP-bound state (active). The switch between the GTP-GDP bond states is controlled by accessory proteins: (1) the guanine nucleotide exchange factors (GEFs), which promote the exchange of GDP for GTP; (2) the GTPases activating proteins (GAPs), which accelerate the hydrolysis of GTP. Therefore, it’s important for biosensor to monitor the both sides simultaneously. Fortunately, LFCA are completely reversible^27^, allowing to detect not only the exchange of GDP to GTP (Ras activation) but also the subsequent hydrolysis of GTP (Ras inactivation). This merit was also conformed in our experiment using Gefitinib to reverse the effect of EGF stimulation or EGF to cripple the inhibition of Gefitinib. The exchange of GDP to GTP induce the decrease of bioluminescence, whereas, the hydrolysis of GTP increases the bioluminescence. In the continuous biological processes of living cells, the interconversion between GDP and GTP is in dynamic balance. And the activity change of the Raf-Fluc biosensor was due to a shift in the Ras-GDP/Ras-GTP balance.

## Conclusions

In conclusion, we have developed a novel monomolecular bioluminescent Ras signaling biosensor based on luciferase fragment complementation strategy. This biosensor allows non-invasive, real time, dynamic and reversible imaging as well as quantification of endogenous Ras activity in living cells and *in vivo*. We showed that the Raf-Fluc gives the robust and sensitive reporting efficiency in response to Ras pathways stimulation and inhibition. The only flaw of the Raf-Fluc is the challenge of lentivirus packaging due to long gene sequences. With the potential ability to monitor the efficacy of targeting Ras pathways, the selected cell lines stably carrying the Raf-Fluc gene will further extends its application spectrum. This innovative work opens up the way for the identification of lead compounds targeted Ras signaling from libraries using cell-based, high-throughput screening, and the evaluation of the efficacies in preclinical models.

## Materials and Methods

### Chemicals, enzymes and reagents

Restriction enzymes and DNA ligase were purchased from Fermentas (Thermo Fisher Scientific Inc. Waltham, USA). TransStart FastPfu DNA polymerase was obtained from TransGen Biotech (Beijing, CN). ClonExpress^TM^ MultiS One Step Cloning kit was obtained from Vazyme Biotech (Nanjing, CN). Plasmid extraction kits and DNA gel extraction kits were purchased from Qiagen (Valencia, CA). LipoFiter^TM^ transfection reagent was purchased from Hanbio Biotech (Shanghai, CN). TRIzol reagent was obtained from Invitrogen (Carlsbad, CA, USA). EGF was purchased from peprotech (Rocky Hill, NJ, USA). Gefitinib was from AstraZeneca (London, UK). D-Luciferin potassium was from Xenogen (Alameda, CA). Coelenterazine was purchased from Regis (Morton Grove, IL, USA).

### Construction of the gene encoding Raf-Fluc

The N-terminal (AA 1~416) and C-terminal (AA 398~550) of *firefly* luciferase (Nluc and Cluc) were amplified by PCR from pGL3-Basic (Promega, #E1751), respectively. The total coding sequences of Raf-1 (Raf) were amplified by RT-PCR from the cDNA library of K562 cells. Gaussia luciferase (Gluc) was amplified by PCR from the pGLuc-Basic (NEB, #E3300). The IRES gene sequences were amplified from the pLVX-IRES-mCherry (Clontech, #631237). These fragments of the biosensor, Nfluc-Raf-Cfluc-IRES-Gluc, was orderly and seamlessly constructed using ClonExpress^TM^ MultiS One Step Cloning kit into pcDNA3.1(+) vector. The flow chart for constructing the sensor was outlined in Fig. 1. The mutants: Raf-Fluc(C168S), H-Ras(G12V) and H-Ras(N116Y) were constructed using the appropriate primers and the QuickChange^TM^ site-directed mutagenesis kit (Stratagene, #200518).

### Cell Culture

Human colorectal cancer SW1116 cells and Leukemia K562 cells were cultured in Dulbecco modified Eagle medium (DMEM, Gibco Laboratories, Grand Island, NY) supplemented with 10% fetal bovine serum (Gibco, New York). Cell cultures were maintained in a 37 °C incubator with 5% CO_2_.

### Cells-based in *Vitro* assay

For transfection of SW1116, the plasmids were transfected in 80% confluent 24-h-old cultures on 48-well using LipoFiter^TM^ transfection reagent according to the manufacturer’s instructions. *In vitro* bioluminescent analyses were performed in transfected or cotransfected cells after administration with EGF or (and) Gefitinib and incubation for the indicated times. Each treatment was done in triplicate. *Gaussia* and *firefly* luciferase bioluminescence imaging were obtained in the same living cells. The measure of *gaussia* luciferase activity, as the internal control bioluminescence, was preferential to avoid mutual interference, because *gaussia* luciferase emission signal intensity is almost negligible at 600 nm after minutes due to the rapid kinetics of coelenterazine. For the complemented *firefly* activity, after addition of D-luciferin (150 ug/ml in Cell Culture Medium, 100 ul/well), luminescent signal intensity (photons/second/square centimeter/steridian or p/s/cm^2^/sr) was measured by IVIS spectrum (Caliper Life Sciences, Hopkinton, MA) using the following parameters: 15 s exposure; emission filter, 600 nm; f-stop, 1; binning, 8; field of view, 15 cm. For *gaussia* luciferase activity, bioluminescence was measured by adding coelenterazine (1.5 uM in DPBS, 100 ul/well) with IVIS spectrum (30 s exposure; emission filter, 500 nm; f-stop, 1; binning, 8; field of view, 15 cm). Data for each well are expressed in the normalized bioluminescence ratio, which is calculated as the ratio of the luminescent intensity of *firefly* luciferase (Fluc) at 600 nm to that of *gaussia* luciferase (Gluc) at 500 nm (Fluc/Gluc).

### Western Blotting

After corresponding treatment, the transfected cells were harvested in the lysis buffer (150 mM NaCl, 1% NP-40, 50 mM Tris-HCl pH 7.4, 1 mM phenylmethylsulfonyl fluoride, 1 µg/ml leupeptin, 1 mM deoxycholic acid and 1 mM EDTA). The cell lysates was directly subjected to SDS-PAGE and probed with a 1:1000 dilution of goat anti-luciferase polyclonal antibody (anti-luciferase pAb, Promega, catalog #G745A) to confirm the expression of the biosensor. The primary antibody was detected with a 1:2000 dilution of HRP-conjugated donkey anti-goat IgG (Promega, catalog #V805A). Blots were developed using enhanced chemiluminescence (ECL) reagent (Amersham BioSciences). Images were gathered by Alpha Innotech’s FluorChem imaging system (Alpha Innotech Corp., San Leandro, CA, USA).

### GST pull-down assay

SW1116 cells on 100 mm culture dishes were transfected with 4 ug Raf-Fluc. After treatment with 40 ng/ml EGF or 5 nM Gefitinib for 30 min, the cells were then harvested by the lysis buffer (20 mM Tris, pH 7.5, 150 mM NaCl, 1% TritonX-100, 2.5 mM sodium pyrophosphate, β-glycerophosphate, 1 mM EDTA, 1 mM Na_3_VO_4_, 1 ug/ml leupeptin, 1 mM phenylmethylsulfonyl fluoride). The Active Ras Pull-Down and Detection Kit (Thermo, #16117) was used to do the pull-down assay, according to the manufacturer’s instructions.

### In *Vivo* mouse imaging experiments using xenografts

All experimental procedures with animals used in this study was approved by the Experimental Animal Manage Committee of Sichuan University. Animal handling and all procedures on animals were carried out strictly according to the guidelines of the Animal Care and Use committee of Sichuan University and the Animal Ethics Committee Guidelines of the Animal Facility of the West China Hospital. The 5-week-old female nude mice were maintained under specific pathogen free (SPF) conditions. SW1116 cells transfected with 5 ug Raf-Fluc were implanted subcutaneously 24 h later (about 5 × 10^7^ cells). The bioluminescence of *firefly* and *gaussia* luciferases were obtained from the same xenograft with different emission filters after a residence time of 24 h The measure of *gaussia* luciferase activity was preferential to avoid mutual interference. For *firefly* luciferase bioluminescence, the mice were imaged after i.p. injection of D-luciferin (150 mg/kg BW) using the following parameters: 2-min exposure; emission filter, 600 nm; f-stop, 1; binning, 8; field of view, 15 cm. For *gaussia* luciferase activity, bioluminescence was measured after i.p. injection of coelenterazine (1 mg/kg BW) with the parameters: 3 min exposure; emission filter, 500 nm; f-stop, 1; binning, 8; field of view, 15 cm.

## Electronic supplementary material

Supplementary Info File
