# Supplementary material for: Noninvasive Imaging of Ras Activity by Monomolecular Biosensor Based on Split-Luciferase Complementary Assay
Source: Sci Rep. 2017 Aug 30;7:9945. doi: 10.1038/s41598-017-08358-3 (PMC5577193; doi:10.1038/s41598-017-08358-3)
Supplement: Supplementary file 1 — Supplementary Info File [file 41598_2017_8358_MOESM1_ESM.pdf]

Noninvasive Imaging of Ras Activity by Monomolecular Biosensor Based on Split-Luciferase Complementary Assay

Liang Chen [a,#], Wei Bing Leng [a,b,#], De Zhi Li [a,b], Hong Wei Xia [a], Min Ren [a,b], Qiu Lin Tang [a], Qi Yong Gong [c], Fa Bao Gao [c], Feng Bi [a,b,*]

[a] Laboratory of Molecular Targeted Therapy in Oncology, State Key Laboratory of Biotherapy, Sichuan University, Chengdu, Sichuan, China
[b] Department of Medical Oncology, West China Hospital, Sichuan University, Chengdu, Sichuan, China
[c] Department of Radiology, West China Hospital, Sichuan University, Chengdu, Sichuan, China

[*] Corresponding author at: Laboratory of Molecular Targeted Therapy in Oncology, State Key Laboratory of Biotherapy, Sichuan University, Chengdu, Sichuan, China.
Tel: +86-28-85423609; fax: +86-28-85164046
E-mail address: bifeng@medmail.com.cn

[#] These authors contributed equally to the work.

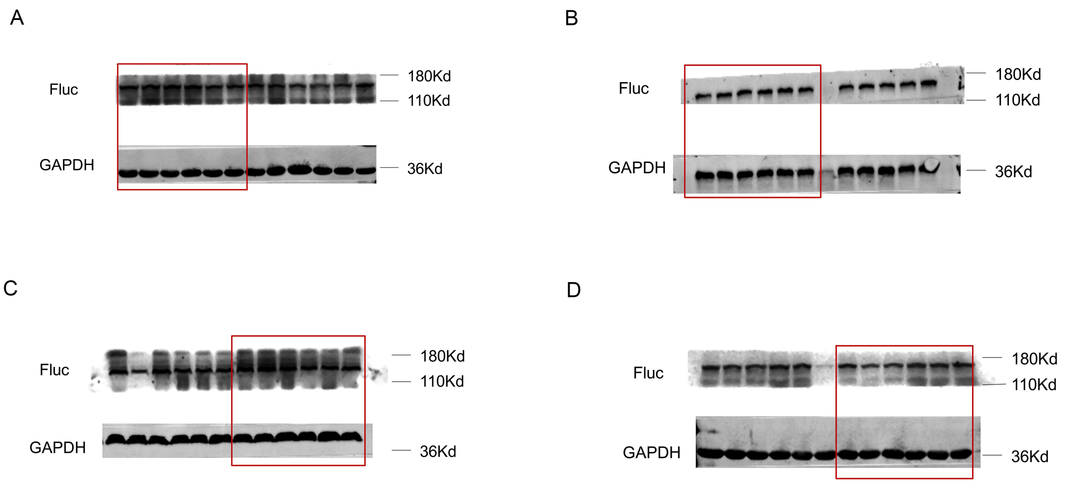

Figure S1. Full-length blot images for Figure 3.

Fig.S1A: full Western blot of Fig. 3A .

Fig. S1B: full Western blot of Fig. 3B.

Fig. S1C: full Western blot of Fig. 3C.

Fig. S1D: full Western blot of Fig. 3D.

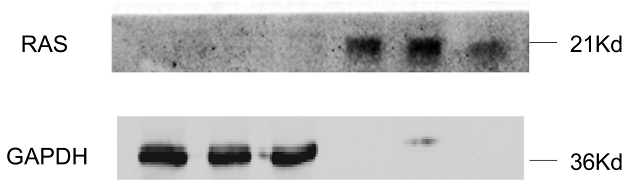

Figure S2. Full-length blot images for Figure 4.
